# Smartphone-Based Digital Eczema Education Program for Atopic Dermatitis in Children Aged 0 to 6 Years: Multicenter, Randomized, Parallel Controlled Clinical Study

**DOI:** 10.2196/79559

**Published:** 2026-01-07

**Authors:** Huan Yang, Hong Shu, Liu-hui Wang, Ping Li, Yun-ling Li, Qin-feng Li, Xiu-ping Han, Jing Tian, Jing Chang, Hua Qian, Jing-ping Chen, Xin-qiang Ding, Pan-qian Wu, Li-min Dou, Zhen Luo, Wei Li, Yang-yang Lin, Lin Li, Shu-zhen Yue, Yang Gu, Li Yang, Xiao-hong Sun, Xiao-yan Luo, Lin Ma, Hua Wang

**Affiliations:** 1 Department of Dermatology, Children’s Hospital of Chongqing Medical University, National Clinical Research Center for Child Health and Disorders, Ministry of Education Key Laboratory of Child Development and Disorders, NO.136 Zhongshan 2nd Road, Yuzhong District, Chongqing, 400014, China, +86-23-63638830; 2 Department of Dermatology, Kunming Children's Hospital, Kunming, China; 3 Department of Dermatology, Children’s Hospital of Fudan University, Shanghai, China; 4 Department of Dermatology, Shenzhen Children’s Hospital, Shenzhen, China; 5 Department of Dermatology, The Children's Hospital, Zhejiang University School of Medicine, National Clinical Research Center of Child Health, Hangzhou, China; 6 Department of Dermatology, Tianjin Children’s Hospital, Tianjin, China; 7 Department of Dermatology, Shengjing Hospital of China Medical University, Shenyang, China; 8 Department of Dermatology, Beijing Children’s Hospital, Capital Medical University, National Center for Children’s Health, Beijing, China; 9 Department of Dermatology, Hunan Children's Hospital, Changsha, China; 10 Department of Dermatology, Children’s Hospital of Soochow University, Suzhou, China; 11 Department of Dermatology, Guangzhou Woman and Children’s Medical Center, Guangzhou, China; 12 Department of Dermatology, Xi'an Children's Hospital, Xi'an, China

**Keywords:** atopic dermatitis, digital education, smartphone-based intervention, caregiver engagement, relapse prevention, randomized controlled trial, pediatric dermatology, telemedicine portal, self-management, multicenter study

## Abstract

**Background:**

Atopic dermatitis (AD) is a chronic, relapsing inflammatory skin condition that affects approximately 10% to 20% of children, imposing substantial health and economic burdens. Although education for patients and caregivers is acknowledged as a crucial element in the management of AD, conventional approaches, such as workshops or in-person consultations, are often resource intensive and face challenges related to scalability, personalization, and relapse prevention. Digital tools present promising alternatives; however, empirical evidence supporting their effectiveness in young children is currently limited.

**Objective:**

This study aimed to evaluate whether a smartphone-based patient-caregiver educational program could reduce relapse rates in children aged 0 to 6 years with moderate-to-severe AD, compared with conventional outpatient consultation alone.

**Methods:**

In this multicenter, randomized, parallel-controlled trial, 615 children were enrolled across 12 tertiary pediatric dermatology centers in China and randomized (1:1) to receive either a smartphone-based digital education program with standard care (intervention group) or conventional face-to-face consultation only (control group). The 12-week digital program, delivered via the WeChat-based *Skin Care E-Station* platform, included structured multimedia modules, interactive educational materials, and a dynamic electronic action plan tailored to the child’s age and disease stage. The primary endpoint was the 12-week relapse rate after the acute treatment phase. The secondary endpoints included changes in disease severity (Scoring Atopic Dermatitis, Peak Pruritus Numerical Rating Scale, and Patient-Oriented Eczema Measure) and quality of life (Children’s Dermatology Life Quality Index or Infant’s Dermatitis Quality of Life Index and Dermatitis Family Impact) up to 52 weeks.

**Results:**

Among 615 randomized participants (mean age 3.3, SD 1.7 y; n=317, 51.5% male), relapse at 12 weeks occurred significantly less frequently in the digital education group than in the control group (16.6% vs 24.0%; relative risk 0.69, 95% CI 0.50‐0.96; *P*=.02). Kaplan-Meier analysis showed superior relapse-free survival over the first 100 days (hazard ratio 0.688, 95% CI 0.490‐0.966; *P*=.03). Differences in relapse rates beyond 12 weeks and in secondary outcomes were not statistically significant. Engagement tracking indicated high adherence to the intervention, with 58.0% of caregivers maintaining regular weekly use of the digital platform.

**Conclusions:**

A structured smartphone-based patient-caregiver educational intervention significantly reduced short-term relapse risk among young children with moderate-to-severe AD, likely through improved caregiver recognition and early management of disease flares. Although effects diminished beyond 12 weeks, this approach demonstrates that scalable digital education is a feasible and effective adjunct to standard care in pediatric AD. Future research should focus on sustaining engagement, optimizing long-term reinforcement, and assessing cost-effectiveness in diverse caregiver populations.

## Introduction

Atopic dermatitis (AD) is a chronic inflammatory skin condition characterized by intense pruritus and eczematous lesions, accompanied by episodic exacerbations and persistent skin symptoms [1]. AD is recognized as a global health concern, affecting up to 20% of children and approximately 3% of adults worldwide [2]. In China, the prevalence of AD stands at approximately 30.48% among infants aged <1 year [3] and 12.94% among children aged 1 to 7 years [4], imposing substantial economic and public health burdens [5,6]. The recurrent nature of AD presents unique challenges, as patients often experience periods of exacerbation interspersed with phases of relative remission [1]. This cyclical pattern highlights the necessity for effective management strategies that not only address current symptoms but also equip patients and their caregivers with the knowledge and skills to prevent future recurrences [7].

Patient-caregiver education has emerged as a pivotal component of AD management, as highlighted in many international guidelines [8-12]. It is important to note that for pediatric AD populations, the primary focus of education is often on parents and caregivers, especially for infants and young children. As children grow older and their understanding develops, education can be progressively tailored and delivered directly to the patients themselves, in an age-appropriate manner. Providing patients and their caregivers with comprehensive information about the condition, its triggers, management strategies, and the importance of adherence to treatment regimens can empower them to take an active role in their own care [13]. Studies have shown that patients and families who receive structured educational programs are more likely to engage in treatment and preventive measures, leading to better overall outcomes and reduced disease severity, as evidenced by metrics such as Scoring Atopic Dermatitis (SCORAD) and Children’s Dermatology Life Quality Index (CDLQI) [14]. Furthermore, patient-caregiver education may contribute to interrupting the “atopic march” toward comorbidities such as allergic rhinitis [15]. Despite the recognized importance of education, gaps remain in the current approaches used to educate patients with AD and their caregivers. Existing educational models, including workshops [16-19], eczema schools [20-23], and printed materials [19,24-26], are resource intensive, lack personalization, or fail to sufficiently address the specific concerns and experiences of patients and caregivers. As a result, patients and their families may feel overwhelmed or unsupported in managing their condition, leading to suboptimal adherence and an increased risk of recurrent flares [27,28]. In addition, prior investigations have predominantly focused on the short-term efficacy of educational interventions, whereas the impact of education on the prevention of AD relapse remains considerably underexplored [29].

The COVID-19 pandemic has instigated a paradigm shift toward digital health [30], resulting in an increased willingness among patients with AD and caregivers to use digital tools. Mobile platforms that integrate multimedia content, such as videos, interactive texts, and illustrated narratives, offer advantages in accessibility, engagement, and personalized learning experiences. These platforms theoretically address traditional limitations by providing scalable and adaptive interventions. However, empirical evidence supporting such innovations remains limited, particularly in children with AD.

In this multicenter, randomized controlled trial (RCT), we aim to assess the efficacy of a smartphone-based digital educational program in comparison to conventional outpatient consultation alone on the relapse rates of AD. By leveraging high smartphone penetration, this trial seeks to develop a more effective educational intervention that will ultimately reduce the burden of AD.

## Methods

### Study Design

This multicenter, parallel RCT (ChiCTR2000031474) was conducted within the pediatric dermatology departments of 12 tertiary public hospitals across China, including locations in Chongqing, Beijing, Liaoning, Shanghai, Shenzhen, Zhejiang, Hunan, Xi’an, Suzhou, Tianjin, Kunming, and Guangdong (Multimedia Appendix 1). The trial adhered to the CONSORT (Consolidated Standards of Reporting Trials) reporting guidelines (Checklist 1).

### Participants

Children aged 0 to 6 years diagnosed with AD according to the American Academy of Dermatology Consensus criteria were eligible for inclusion if they presented with moderate-to-severe AD (SCORAD ≥25; Investigator’s Global Assessment [IGA] ≥3) and had caregivers proficient in reading Chinese characters and using a smartphone. All participants were recruited through in-person clinical visits. Exclusion criteria included children with severe infections, psychiatric disorders, primary or secondary immune deficiencies, malignancies, or other medical conditions that significantly impair quality of life. Additionally, caregivers with mental disorders or cognitive impairment, prior participation in any patient-caregiver education program, and severe AD requiring systemic treatment were excluded.

Most caregivers were the patients’ parents, and a minority were grandparents. All caregivers lived in the same household as the child, as families of “left-behind children,” defined as children who remain in their hometowns in China for more than 6 months under the care of grandparents or other relatives while both parents migrate to urban areas for work, were excluded to avoid potential confounding due to social and environmental separation. Caregiver demographic details (eg, age and education level) were not collected, as the original study design focused on patient-level outcomes.

### Randomization and Blinding

Eligible patients were randomly assigned in a 1:1 ratio to either the intervention group (smartphone-based digital education program plus standard care) or the control group (conventional outpatient consultation only). Randomization was conducted using a computer-generated number table with a block size of 4. The digital educational intervention protocol was developed and administered through a secure cloud-based platform operated by an independent clinical research organization (CRO) not involved in trial execution. This third-party CRO was responsible for maintaining the randomization database, delivering standardized digital educational content (including interactive modules, video tutorials, and self-assessment tools), and monitoring intervention adherence by automated adherence reminders (SMS text messaging or telephone) and digital footprint tracking (platform logins and content interaction time). Crucially, the CRO had no involvement in participant recruitment, clinical management, or outcome evaluation processes to ensure blinding integrity.

### Intervention and Procedures

#### Overview

Before trial initiation, all participating dermatologists completed a 4-hour standardized training program on evidence-based patient-caregiver education.

At baseline enrollment, all patients were provided with a 1-page education leaflet (Multimedia Appendix 2) and completed disease severity assessments along with standardized questionnaires. Subsequently, participants entered a 2-week acute-phase treatment period, conducted strictly according to established clinical guidelines [10-12]. The acute-phase regimen included once-daily topical pharmacotherapy, 0.05% desonide or 0.1% hydrocortisone butyrate for those aged 0 to 2 years and 0.1% mometasone furoate for children older than 2 years, combined with nonpharmacological care. The nonpharmacological regimen consisted of twice-daily application of a ceramide-dominant emollient and daily bathing restricted to fewer than 10 minutes at water temperatures below 38°C. Patient status was reassessed every 14 (SD 2) days using the IGA. Patients achieving an IGA score ≤2 were advanced to the maintenance phase.

#### Digital Education Intervention

**Figure 1.**
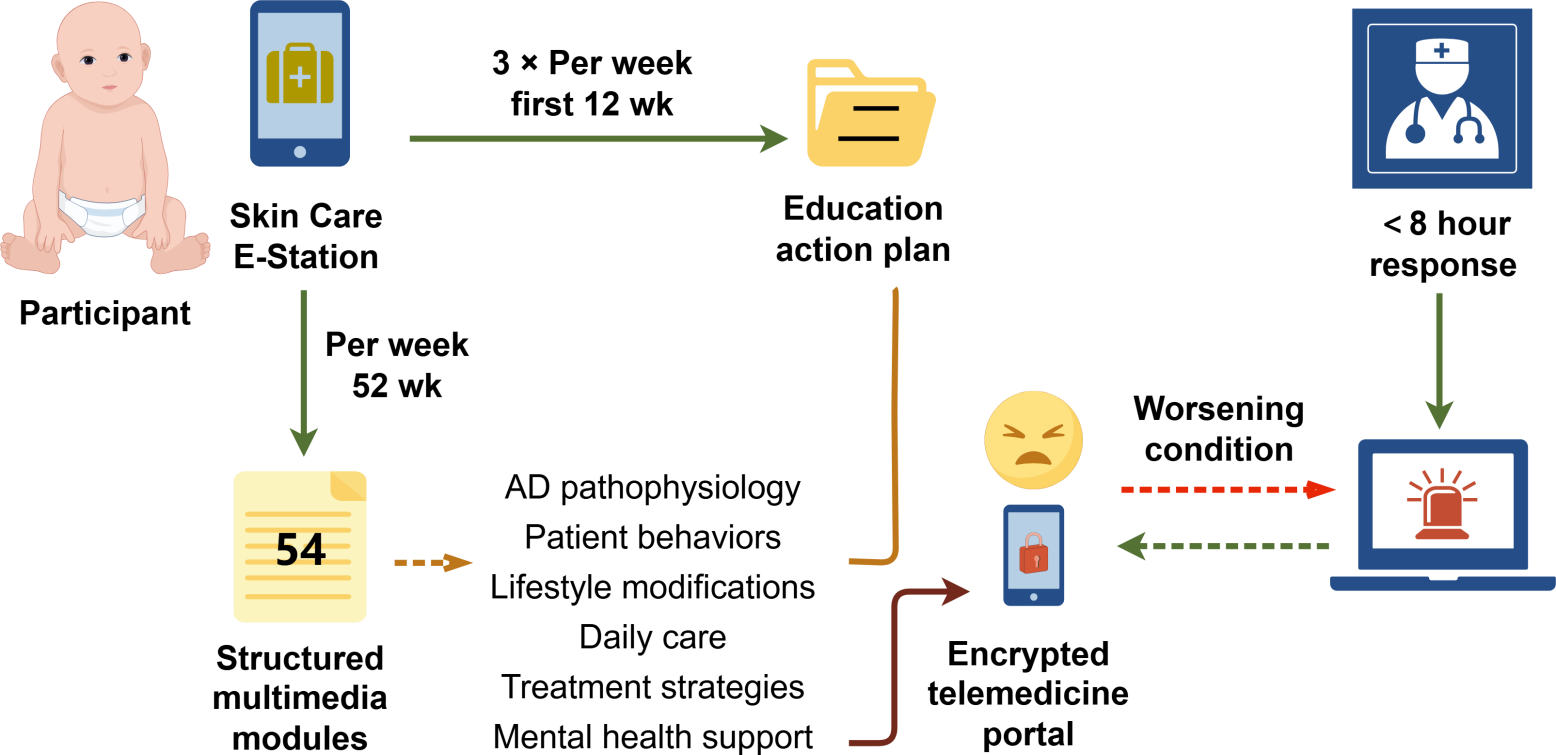
Skin Care E-Station workflow. Participants in the digital education group accessed the clinical research organization–managed smartphone platform, receiving thrice-weekly education action plans (Mondays/Wednesdays/Fridays) for 12 wk alongside 54 structured multimedia modules (text/images/video/animations) distributed evenly throughout the 52-week program. The content covered AD pathophysiology, care routines, treatments, lifestyle, and mental health support, with real-time clinician access via an encrypted telemedicine portal (<8 h response) during flares. The illustration was created on the FIGDRAW 2.0 platform. AD: atopic dermatitis.

Upon entering the maintenance phase, participants in the intervention group were instructed to receive the digital health education program via the WeChat (Tencent Holdings Ltd)–based online platform “Skin Care E-Station” (Figure 1). This digital portal contained all educational components and could be accessed by scanning a QR code, without requiring any additional software installation. A procedural overview of patient enrollment and identity verification within the platform is provided in Multimedia Appendix 3. As WeChat is a widely used multifunctional communication, social, and payment application in China, this ensured high accessibility and user engagement. The platform consisted of three core components: (1) structured multimedia educational modules, (2) automated follow-up reminders, and (3) emergency response protocols for disease exacerbations.

The digital education comprised 2 major elements. The first element was an electronic education action plan (EAP). Each participant in the intervention group received a digital, dynamically tailored EAP (Multimedia Appendix 4), customized according to the child’s age group (1‐2 y vs 2‐6 y) and disease phase (acute flare vs maintenance). The EAP provided phase-specific guidance on bathing routines, emollient application, medication use, self-assessment of disease severity, and access to emergency consultations. The EAP was automatically delivered 3 times per week (Mondays, Wednesdays, and Fridays) for 12 consecutive weeks. The second element was structured multimedia educational modules. The core educational curriculum was collaboratively developed by the 12 participating centers, integrating an analysis of the most frequent caregiver concerns and key clinician recommendations from routine practice, supplemented by insights and experiences from other research teams [17-24]. This content was organized into 6 domains (AD pathophysiology, patient behaviors, lifestyle modifications, daily care, treatment strategies, and mental health support) and subsequently produced by an independent third-party company into 54 structured multimedia modules. These modules were delivered in formats tailored for children aged 0 to 6 years and their caregivers, including illustrated text (sample in Multimedia Appendix 5), images (sample in Multimedia Appendix 6), videos (sample in Multimedia Appendix 7), and animated stories (sample in Multimedia Appendix 8). The intervention group did not receive any additional systematic face-to-face patient education during scheduled follow-up visits or at unscheduled clinical encounters.

#### Control Group

Participants in the control group received only conventional education, consisting of a 15-minute face-to-face counseling session conducted at each scheduled follow-up visit. They were also provided with access to the “Skin Care E-Station” platform for noneducational purposes, including data collection and automated follow-up reminders, but did not receive any educational content, such as the EAP or structured multimedia modules.

#### Maintenance-Phase Treatment Regimen

Both the intervention and control groups received identical pharmacological treatments during the maintenance phase. In the 0 to 2 years age group, maintenance therapy comprised twice-weekly application of 0.05% desonide or 0.1% hydrocortisone butyrate cream, together with daily emollient use. In children aged >2 years, maintenance consisted of twice-weekly application of 0.03% tacrolimus ointment, alongside regular emollient therapy.

### Assessments and Outcomes

Assessments were conducted at 4, 8, 12, 24, 36, and 52 weeks following randomization. Disease severity was evaluated using SCORAD, IGA, Peak Pruritus Numerical Rating Scale (PP-NRS) for pruritus, and the Patient-Oriented Eczema Measure (POEM). Quality of life was assessed using the Dermatitis Family Impact (DFI), the CDLQI, and the Infant’s Dermatitis Quality of Life Index (IDQOL). The assessments were completed as follows: If the child was able to attend an in-person visit, the evaluations were performed by physicians not involved in the educational intervention; if not, caregivers uploaded photos and completed the PP-NRS, POEM, Quality of Life (QoL) questionnaires, and SCORAD self-assessment (Multimedia Appendix 9) on the platform. Subsequently, physicians not involved in the educational intervention performed the IGA and SCORAD assessments based on the uploaded photos and the SCORAD self-assessment form.

In addition, the timing of each relapse was meticulously recorded. Relapse was defined as an increase of ≥10 points in the SCORAD score relative to the value recorded at the conclusion of the 2-week acute-phase treatment. The primary endpoint was the relapse rate at 12 weeks. The secondary endpoints included the changes in disease severity scores (SCORAD, PP-NRS, and POEM) and quality of life scores (CDLQI/IDQOL and DFI) from week 2 (end of the acute-phase treatment) to weeks 4, 8, 12, 24, 36, and 52 in both groups.

### Statistical Analyses

Statistical analyses were performed using R (version 4.0.5; R Foundation for Statistical Computing). Sample size estimation was based on an expected 12-week relapse rate of 30% in the digital education group and 45% in the control group, which were derived from a multicenter randomized controlled clinical study on long-term intermittent maintenance therapy for children with AD conducted in China [31]. Assuming a 1:1 allocation ratio and a 30% expected dropout rate, an empirically supported adjustment commonly recommended in sample size estimation to maintain statistical power for both completer and intention-to-treat (ITT) analyses [32], a minimum of 229 patients per group was required to achieve 80% power with an α level of .05.

All analyses followed the ITT principle, which includes all randomized participants in the groups to which they were originally assigned. This approach maintains the integrity of randomization and provides a conservative estimate of treatment effectiveness in real-world clinical settings. To address missing data, multiple imputation by chained equations (MICEs) was performed under the assumption that the data were missing at random. Missing at random implies that the probability of missingness depends only on observed data and not on unobserved data. MICEs generate multiple plausible values for missing data based on observed variables, and we used 5 imputations (m=10) to account for uncertainty. The results from the imputed datasets were combined according to Rubin’s rules. Normality of continuous variables was evaluated using the Kolmogorov-Smirnov test, a nonparametric test that compares the empirical distribution of the data with a theoretical normal distribution to assess deviations from normality. Data with normal distributions were presented as mean (SD) and compared using independent *t* tests. Nonnormally distributed data were presented as median (IQR) and analyzed using the Mann-Whitney test.

For the primary outcome, the 12-week relapse rate was compared between groups using the chi-square test. Time to relapse over 100 days was visualized using Kaplan-Meier curves and compared using the log-rank test. Cox proportional hazards regression was used to estimate hazard ratios (HRs) and 95% CIs, both unadjusted and adjusted for age and sex.

For continuous outcomes repeatedly measured over time (SCORAD, POEM, PP-NRS, IDQOL/CDLQI, and DFI), population-averaged generalized estimating equations were used with a Gaussian family, identity link, exchangeable correlation structure, and robust SEs to account for within-subject correlations. Each model was adjusted for baseline score, age, and sex. Missing data in these repeated measures were handled using MICEs (10 imputations), with final estimates combined using Rubin’s rules.

A 2-sided *P* value of .04 was considered statistically significant.

### Ethical Considerations

This study was reviewed and approved by the Medical Research Ethics Committee of the Children’s Hospital of Chongqing Medical University (approval No. 2019-44-1). Written informed consent was obtained from all participants and their guardians before their involvement in the study. All collected data were deidentified to protect participant privacy and confidentiality. No compensation was provided to the participants.

## Results

### Participant Flow and Cohort Characteristics

**Figure 2.**
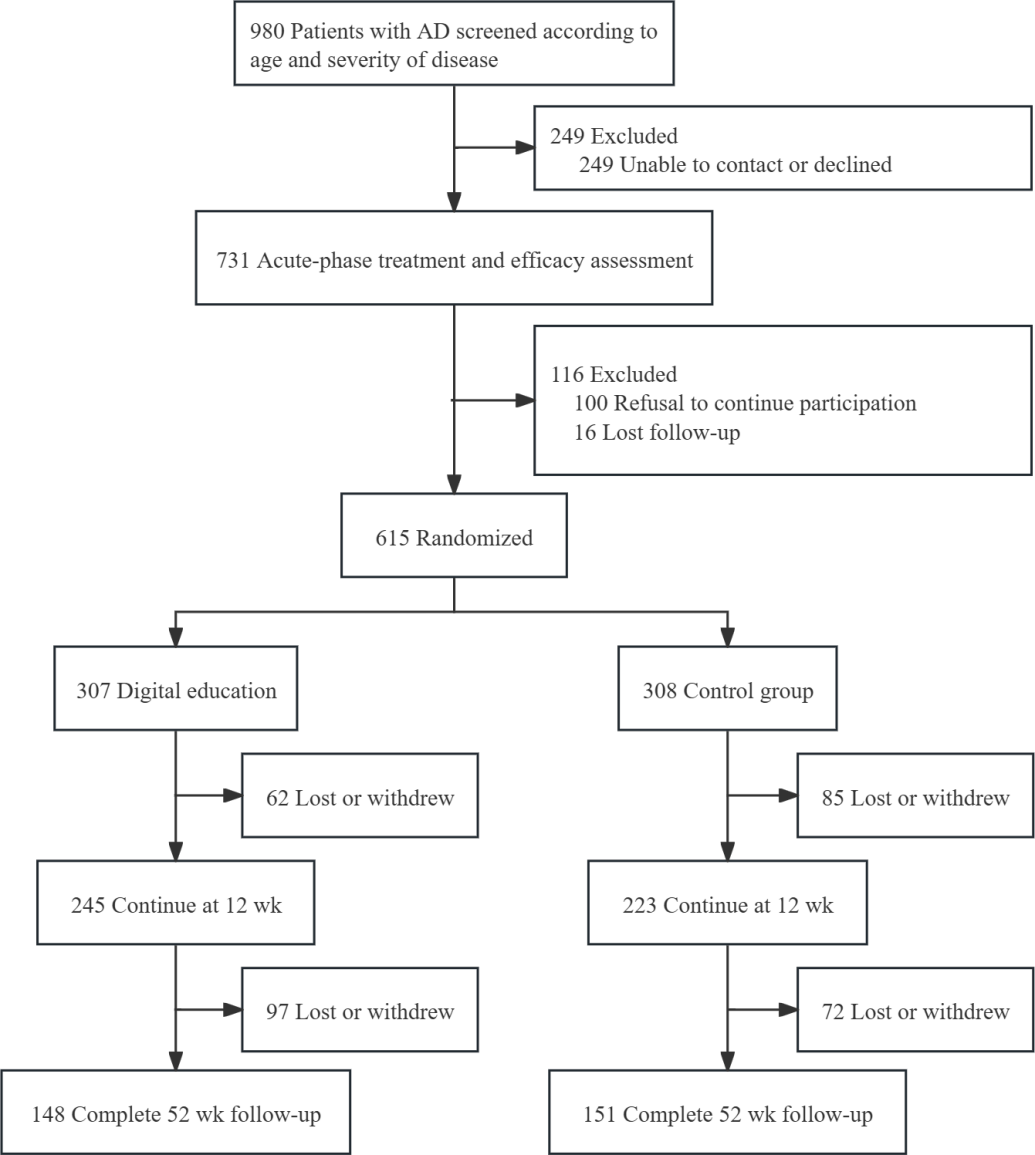
CONSORT diagram of the randomization and follow-up of the study participants. AD: atopic dermatitis.

The multicenter trial was conducted across 12 tertiary dermatology centers in China from July 2020 to March 2021, with a detailed CONSORT flow presented in Figure 2. Of 980 screened children with moderate-to-severe AD, 615 (62.8%) met inclusion criteria and were subsequently randomized into the digital education group (n=307, 49.9%) and the control group (n=308, 50.1%). Significant differences in attrition rates were observed at 12 weeks, with 20.2% (62/307) in the digital education group and 27.6% (85/308) in the control group (95% CI 0.66‐0.98; χ²_1_=4.1; *P*=.04). Ultimately, 48.62% (299/615) completed the 52-week follow-up, comprising the per-protocol population: digital education (n=148, 24.1%) and control (n=151, 24.6%).

Baseline characteristics demonstrated adequate randomization balance (Table 1), with no significant differences observed in gender distribution (men: 52.1% vs 50.8%; *P*=.74), mean age (3.2, SD 1.8, vs 3.4, SD 1.7 y; *P*=.21), or disease severity measures, including SCORAD (49 [40, 59] vs 47.5 [37, 55]; *P*=.29), PP-NRS (6 [6, 8] vs 6 [5, 8]; *P*=.31) and POEM (15 [11, 20] vs 15 [11, 19]; *P*=.50). Similarly, no significant differences were noted in quality-of-life measures, including DFI (9 [4, 16] vs 10 [5,16]; *P*=.77) and QoL scores (13 [11, 15] vs 14 [11, 16]; *P*=.11). Quality of life was assessed using the CDLQI for children and IDQOL for infants.

**Table 1.** The details of clinical characteristics of randomized patients. QoL was assessed using the Children’s Dermatology Life Quality Index for children and the Infants’ Dermatitis Quality of Life Index for infants.

Characteristic	Digital education (n=307)	Control (n=308)	*P* value
Gender, male (%)	52.1	50.8	.74
Age (y), mean (SD)	3.2 (1.8)	3.4 (1.7)	.21
SCORAD^a^, median (Q1^b^ ,Q3^c^)	49 (40, 59)	47.5 (37, 55)	.29
PP-NRS^d^, median (Q1, Q3)	6 (6, 8)	6 (5, 8)	.31
DFI^e^, median (Q1, Q3)	9 (4, 16)	10 (5, 16)	.77
POEM^f^, median (Q1, Q3)	15 (11, 20)	15 (11, 19)	.50
QoL^g^, median (Q1, Q3)	13 (11, 15)	14 (11, 16)	.11

a SCORAD: Scoring Atopic Dermatitis.

b Q1: the first quartile.

c Q3: the third quartile.

d PP-NRS: Peak-Pruritus Numerical Rating Scale.

e DFI: Dermatitis Family Impact.

f POEM: Patient-Oriented Eczema Measure.

g QoL: quality of life.

### Intervention Adherence and Participant Engagement

Intervention adherence, as measured by digital footprint tracking, demonstrated that 58.0% (178/307) of participants in the digital education group maintained regular engagement, averaging 1.64 (SD 0.38) sessions per week, and 26.7% (82/307) accessed the expedited physician consultation portals during disease flares. In the control arm, clinic attendance at the 52-week follow-up was 49.0 % (151/308). The 9.0% absolute difference in adherence between groups was statistically significant (Z=2.3, 95% CI 1.15%-16.85%; *P*=.03).

### Primary Outcome: Relapse Prevention

The ITT analysis revealed a significant reduction in relapse rates in the digital education group at 12 weeks (16.6% [51/307] vs 24.0% [74/308]; relative risk [RR] 0.69, 95% CI 0.50‐0.96; χ²_1_=5.1; *P*=.02). However, there was no statistically significant difference between the 2 groups at other time points: 4 weeks (8.79% vs 12.99%; RR 0.68, 95% CI 0.42‐1.09; χ²_1_=2.8; *P*=.10), 8 weeks (15.64% vs 21.43%; RR 0.73, 95% CI 0.52‐1.02; χ²_1_=3.4; *P*=.07), 24 weeks (21.82% vs 27.60%; RR 0.79, 95% CI 0.60‐1.04; χ²_1_=2.8; *P*=.10), 36 weeks (23.13% vs 29.22%; RR 0.79, 95% CI 0.60‐1.03; χ²_1_=3.0; *P*=.09), and 52 weeks (24.76% vs 31.17%; RR 0.79, 95% CI 0.62‐1.03, χ²_1_=3.1; *P*=.08).

**Figure 3.**
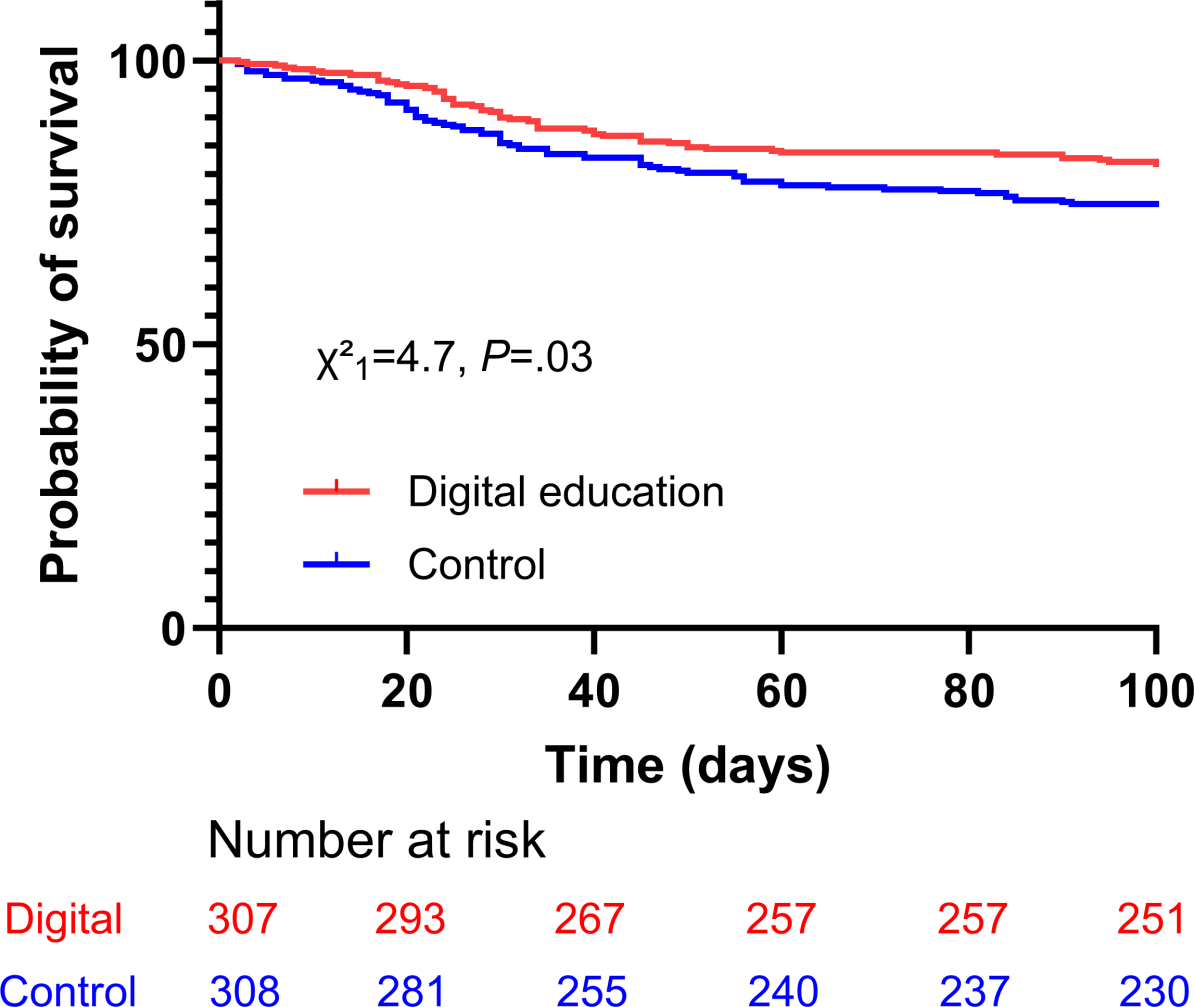
Kaplan-Meier estimates of relapse. Hundred-day relapse-free survival curves in the intention-to-treat set analysis.

Kaplan-Meier analysis (100-d follow-up) further supported the 12-week finding, showing a significant difference in relapse risk between groups (HR] 0.688, 95% CI 0.490‐0.966; χ²_1_=4.7; *P*=.03; Figure 3). In a Cox proportional hazards model adjusting for age and sex, the HR remained significant (adjusted HR 0.665, 95% CI 0.469‐0.943; *P*=.02).

### Secondary Outcomes: Disease Severity Trajectories

Changes in disease severity scores (including SCORAD, PP-NRS, and POEM) and QoL scores (IDQOL/CDLQI and DFI) from week 2 (the end of the acute treatment phase) were compared between the 2 groups at weeks 4, 8, 12, 24, 36, and 52. Using population-averaged GEE models adjusted for baseline score, age, and sex, the results demonstrated no statistically significant differences between the groups in the magnitude of score changes at any of the predefined follow-up time points (Multimedia Appendix 10).

## Discussion

### Principal Findings

This multicenter RCT demonstrated that a structured, smartphone-based multimodal digital education program for caregivers, when integrated with standard care, significantly reduced the substantial proportion of early relapses in children aged 0 to 6 years with moderate-to-severe AD, compared to conventional outpatient consultation alone. This early benefit was further supported by Kaplan-Meier survival analysis showing a clearer separation of relapse-free curves during the early follow-up period. Consistent with the study’s primary objective, these results confirm that the digital educational intervention effectively achieved short-term relapse prevention. However, differences in relapse rates appeared to diminish over time, and no notable differences were observed in secondary end points assessing disease severity or quality of life across the 52-week follow-up.

The observed reduction in early relapse aligns with the core principle of AD management, which emphasizes the critical role of effective patient and caregiver education in improving clinical outcomes [8-12]. The success of our digital program likely stems from its multimodal design, which combined frequent, brief multimedia modules with a “Real-Time Clinician Access” pathway (“Green Channel”). This combination probably enhanced caregivers’ ability to detect early signs of worsening and initiate prompt interventions, thereby preventing progression to full-blown relapse, even in the absence of measurable changes in objective severity indices. The platform’s adherence rate suggested better engagement compared with what is often observed in traditional formats [33], indicating improved feasibility and user engagement.

Our findings are consistent with prior evidence demonstrating that structured education improves short-term management and adherence in pediatric AD [16-23,34-37]. However, they also highlight a common challenge: achieving long-term disease modulation with brief interventions is difficult. The diminishing effect on relapse prevention after the initial 12 weeks may be attributed to several factors. First, the structured program builds knowledge and habits, but its impact may wane without ongoing reinforcement [38]. Second, the natural relapsing-remitting course of AD may eventually overshadow the effects of a time-limited intervention. Furthermore, despite good initial adherence, participant engagement with digital health interventions often declines over time [39].

A critical component contributing to the early success was the integrated “Real-Time Clinician Access,” used by a notable portion of participants during flares or uncertainty. This feature provided timely guidance, effectively replicating the benefit of rapid in-person intervention during exacerbations without the associated logistical burdens. This mechanism, improved early recognition and intervention facilitated by accessible education and on-demand clinical support, provides a plausible explanation for the significant reduction in early relapse rates, underscoring that the primary value of the intervention lies in optimizing the behavioral and self-management aspects of AD care.

The absence of significant differences in disease severity and QoL scores suggests that while the intervention improved caregivers’ short-term flare management, it did not fundamentally alter the underlying core inflammatory process within the assessed follow-up period. This distinction has practical implications: digital education may be most valuable as a tool to reduce short-term exacerbations and health care utilization, complementing rather than replacing ongoing medical and pharmacological strategies needed to modify underlying disease activity.

Traditional educational formats (face-to-face counseling [34-37], workshops [16-19], and eczema schools [20-23] are effective but face significant scalability challenges due to their resource-intensive nature, geographical barriers, and time constraints for families and clinicians [35]. Passive educational materials, such as leaflets and videos, are limited by a lack of interactivity and personalization [26,36,37]. Our study addresses these limitations by leveraging widespread smartphone access to deliver a scalable, multimodal intervention. The platform’s use of brief, engaging modules (animated stories, videos, and illustrated texts) delivered 3 times weekly made essential educational topics accessible to caregivers with varying literacy levels and busy schedules.

### Limitations

Several limitations must be acknowledged. First, smartphone proficiency was an enrollment requirement, potentially excluding digitally underserved groups (eg, from rural or socioeconomically disadvantaged backgrounds) and limiting the generalizability of our findings to urban or digitally connected families. Future implementations should consider hybrid delivery models combining digital modules with printed materials or in-person sessions to enhance inclusivity. Second, understanding of the educational materials was not formally assessed, which may influence outcomes. Third, although early retention differed between groups, a considerable number of participants did not complete long-term follow-up, potentially affecting the accuracy of long-term outcome estimates. Fourth, although outcome assessors were blinded, caregivers and clinicians were not, creating potential for performance or reporting bias. Fifth, caregiver demographic variables (eg, age, education, and occupation) were not collected, limiting our ability to explore effect modification by caregiver characteristics. While the exclusion of ‘left-behind children’ and the predominance of parent caregivers likely reduced heterogeneity in living arrangements and direct caregiving availability, unmeasured socioeconomic or educational differences could still influence the uptake and use of digital education. Finally, while the control group also had access to the platform for noneducational purposes (eg, data entry, random allocation, and scheduling visits), they did not receive the structured educational modules or ‘Green Channel’ clinician access. Therefore, the observed effect likely reflects the combined impact of the educational content and the interactive digital delivery modality, rather than platform access alone. Future trials should include comparative arms using alternative digital formats (eg, app-based platforms and interactive chatbots) to disentangle which specific features most strongly influence adherence and relapse outcomes.

### Conclusions and Broader Implications

In conclusion, this large multicenter RCT demonstrates that a structured, smartphone-based digital education program for caregivers, featuring interactive modules and real-time clinician support, can significantly reduce early relapse rates in young children with moderate-to-severe AD. This finding indicates that scalable digital education can strengthen short-term relapse prevention by optimizing caregiver empowerment and flare management, a strategy particularly relevant in regions with high smartphone penetration.

The broader implications of our study are 3-fold. First, given the high burden of AD and the scalability of digital tools, such interventions hold promise for improving access to quality education and potentially reducing health care disparities. Second, the lower dropout rate in the intervention group suggests that well-designed digital platforms may improve retention in long-term pediatric dermatology research and care. Finally, the transient nature of the benefit underscores that digital education should be viewed as a complementary adjunct to, not a replacement for, ongoing medical management.

Future research should prioritize (1) developing hybrid models that integrate digital education with targeted in-person support to enhance emotional connection and engagement; (2) designing strategies for sustained engagement, such as “booster” modules and adaptive content; (3) ensuring digital accessibility for underserved populations to address health equity; (4) conducting rigorous economic evaluations; and (5) conducting head-to-head comparisons of different digital features and mechanistic studies to identify the active ingredients of digital education.

## Supplementary material

10.2196/79559 Multimedia Appendix 1 List of participating hospitals.

10.2196/79559 Multimedia Appendix 2 Atopic dermatitis awareness material.

10.2196/79559 Multimedia Appendix 3 Procedural overview illustrating the standardized workflow for patient enrollment and identity verification within the “Skin Care E-Station” platform, with representative interface screenshots supporting each operational step.

10.2196/79559 Multimedia Appendix 4 Education action plan.

10.2196/79559 Multimedia Appendix 5 Sample illustrated educational text.

10.2196/79559 Multimedia Appendix 6 Sample caregiver-facing informational images.

10.2196/79559 Multimedia Appendix 7 Sample video.

10.2196/79559 Multimedia Appendix 8 Animated stories.

10.2196/79559 Multimedia Appendix 9 SCORAD self-assessment.

10.2196/79559 Multimedia Appendix 10 Comparison of changes in disease severity and quality of life scores between 2 groups over time.

10.2196/79559 Checklist 1 CONSORT checklist.

## Abbreviations

AD: atopic dermatitis
CDLQI: Children’s Dermatology Life Quality Index
CONSORT: Consolidated Standards of Reporting Trials
CRO: Clinical Research Organization
DFI: Dermatitis Family Impact
EAP: education action plan
HR: hazard ratio
IDQOL: Infant’s Dermatitis Quality of Life Index
IGA: Investigator’s Global Assessment
ITT: intention-to-treat
MICE: multiple imputation by chained equation
POEM: Patient-Oriented Eczema Measure
PP-NRS: Peak Pruritus Numerical Rating Scale
QoL: quality of life
RCT: randomized controlled trial
RR: relative risk
SCORAD: Scoring Atopic Dermatitis
