# Supplementary material for: Smartphone-Based Digital Eczema Education Program for Atopic Dermatitis in Children Aged 0 to 6 Years: Multicenter, Randomized, Parallel Controlled Clinical Study
Source: J Med Internet Res. 2026 Jan 7;28:e79559. doi: 10.2196/79559 (PMC12779099; doi:10.2196/79559)
Supplement: Multimedia Appendix 1 [file jmir-v28-e79559-s001.pdf]

Multimedia Appendix 1

List of Participating Hospitals

1. Department of Dermatology, Children’s Hospital of Chongqing Medical University, Chongqing, China

2. Department of Dermatology, Kunming Children's Hospital, Kunming, China

3. Department of Dermatology, Children’s Hospital of Fudan University, Shanghai, China

4. Department of Dermatology, Shenzhen Children’s Hospital, Shenzhen, China

5. Department of Dermatology, Children's Hospital, Zhejiang University School of Medicine, National Clinical Research Center for Child Health, Hangzhou, China

6. Department of Dermatology, Tianjin Children’s Hospital, Tianjin, China

7. Department of Dermatology, Shengjing Hospital of China Medical University, Shenyang, China

8. Department of Dermatology, Beijing Children’s Hospital, Capital Medical University, National Center for Children’s Health, Beijing, China

9. Department of Dermatology, Hunan Children's Hospital, Changsha, China

10. Department of Dermatology, Children’s Hospital of Soochow University, Suzhou, China

11. Department of Dermatology, Guangzhou Women and Children’s Medical Center, Guangzhou, China

12. Department of Dermatology, Xi'an Children's Hospital, Xi’an, China
