# Supplementary material for: Smartphone-Based Digital Eczema Education Program for Atopic Dermatitis in Children Aged 0 to 6 Years: Multicenter, Randomized, Parallel Controlled Clinical Study
Source: J Med Internet Res. 2026 Jan 7;28:e79559. doi: 10.2196/79559 (PMC12779099; doi:10.2196/79559)
Supplement: Multimedia Appendix 2 [file jmir-v28-e79559-s002.pdf]

Multimedia Appendix 2

Atopic Dermatitis Awareness Material

1. Understanding Atopic Dermatitis

Atopic dermatitis (AD) is a chronic, relapsing inflammatory skin condition. While there is currently no cure, it can be effectively managed with appropriate care and treatment. Multiple factors contribute to the development of AD, including:

- Genetic predisposition—for example, a family history of allergic diseases such as asthma or allergic rhinitis;
- An immature immune system, particularly in infants;
- Defects in the skin barrier;
- Environmental triggers, such as allergens, climate changes, etc.

2. Basic treatment

- Clothing: Choose cotton clothing whenever possible. Avoid scratching and friction against the skin;
- Environment: Maintain a stable indoor temperature and a clean living environment to reduce exposure to allergens like house dust mites, pet dander, and pollen;
- Diet: Monitor food reactions and avoid known food allergens;
- Bathing: Limit bathing to 10 minutes per day with water not exceeding 38°C. Use soap-free cleansers and consider adding bath emollients;
- Moisturizing: Apply emollients to the entire body after bathing, and continue moisturizing 1–2 times daily to maintain skin hydration;
- Avoid scratching. Ensure proper ventilation in the bedroom and avoid overheating.

3. Topical medication therapy

- **Goal:** The goals of treatment for AD are to restore the skin's barrier function, identify and eliminate triggers or aggravating factors, and alleviate symptoms. Always seek care at a certified medical institution and follow treatment guidelines under the supervision of a healthcare professional. Avoid using products not approved by the National Medical Products Administration.
- **First-line treatment:** According to the Chinese Guidelines for the Treatment of Atopic Dermatitis, topical corticosteroids are the first-line treatment. A two-week course of medium-potency corticosteroids is typically effective in controlling flare-ups. For maintenance, apply twice weekly (e.g., Mondays and Thursdays) to prevent rebound or resistance, or switch to a lower-potency preparation. For children, applying a systemic emollient for 15 minutes before corticosteroid ointment enhances efficacy and helps reduce overall corticosteroid use.
- **Second-line treatment:** Calcineurin inhibitors (e.g., pimecrolimus or tacrolimus) or phosphodiesterase 4 (PDE4) inhibitors (e.g., crisaborole) used sequentially or in combination after corticosteroid-induced remission.

4. Use of Topical Corticosteroids

- In children, avoid applying corticosteroids to large areas at once. The total monthly dose should be less than 30 grams. Dosing can be estimated using "fingertip units"—a strip of ointment from the fingertip to the first knuckle covers an area roughly the size of two adult palms.
- Strong corticosteroids should not be used for more than 2 weeks continuously, while mild to moderate preparations should be limited to 4 weeks. On delicate skin areas (e.g., face, armpits, groin), avoid occlusive dressings to prevent excessive absorption.
- Apply corticosteroids 1–2 times daily. Do not discontinue treatment without medical advice. Many relapses occur when caregivers stop treatment prematurely. When used correctly, topical corticosteroids are effective and safe. Most side effects arise from inappropriate overuse. Growth suppression is generally associated with long-term high-dose oral corticosteroids. Long-term topical use (over three consecutive months) may cause local skin effects, such as thinning or hyperpigmentation.
