# Supplementary material for: Smartphone-Based Digital Eczema Education Program for Atopic Dermatitis in Children Aged 0 to 6 Years: Multicenter, Randomized, Parallel Controlled Clinical Study
Source: J Med Internet Res. 2026 Jan 7;28:e79559. doi: 10.2196/79559 (PMC12779099; doi:10.2196/79559)

How to Follow “Skin E-Station”

How patients can successfully follow “Skin E-Station”: steps and key notes

Step 1: After meeting the inclusion/exclusion criteria, the doctor creates a patient record in the electronic medical system as shown below:

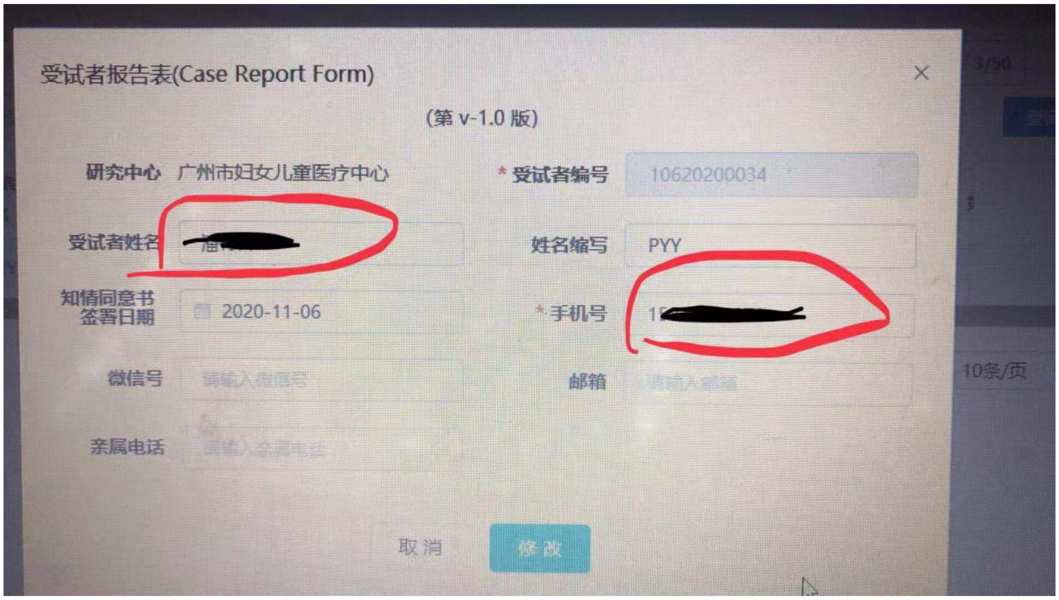

Notes:

1. The participant’s name and mobile number must be accurate. Identity verification later will only succeed if both match exactly. If the child's name is entered incorrectly or the father’s phone number is used during record creation but the mother’s number is used later, verification will fail. Therefore, accuracy during record creation is essential.

2. The patient must not follow the “Skin E-Station” public account before the doctor creates the record; otherwise, identity verification will fail. If this occurs, ask the caregiver to unfollow the account. Once the doctor creates the record, the caregiver should scan the doctor’s QR code for verification and enrollment.

Step 2: After screening, show the doctor’s QR code to the patient

Doctor-side steps on the mobile phone:

1. Follow the “Skin E-Station” public account

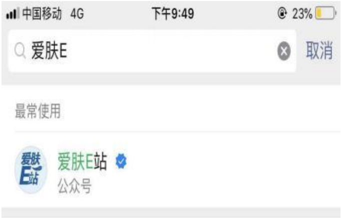

2. Tap “Doctor Information” → “Doctor Login”; both the account and password are the doctor’s phone number.

Note: If the password does not work, use SMS verification code to log in.

3. After logging in, tap “Personal Center”.

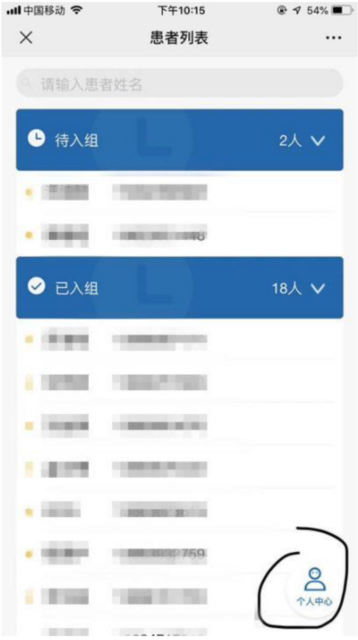

4. Show the QR code to the caregiver.

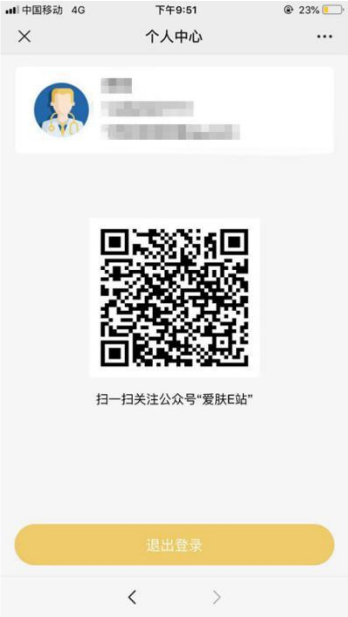

Note: This QR code is dynamic and expires in about 3 days. Once expired, a new QR code must be presented.

Step 3: The caregiver scans the QR code to follow “Skin E-Station”

1. After scanning, the caregiver will immediately receive an identity verification message, as shown below:

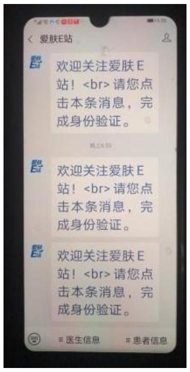

2. The caregiver taps the message to verify identity; the successful verification interface is shown below:

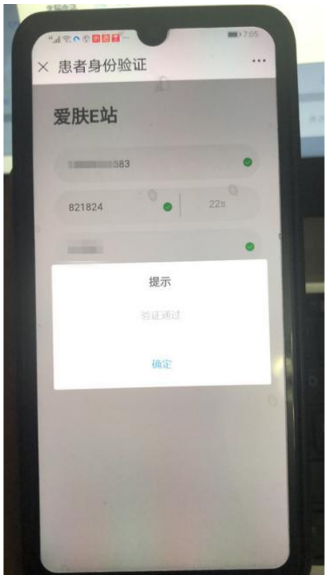

Note: The caregiver must enter the same name and phone number used during record creation.

3. After the caregiver logs in via “Patient Information,” the interface below indicates successful enrollment.

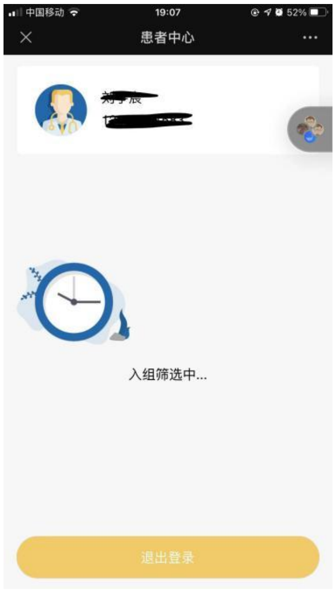

Supplement: Multimedia Appendix 3 [file jmir-v28-e79559-s003.pdf]
