# Supplementary material for: Smartphone-Based Digital Eczema Education Program for Atopic Dermatitis in Children Aged 0 to 6 Years: Multicenter, Randomized, Parallel Controlled Clinical Study
Source: J Med Internet Res. 2026 Jan 7;28:e79559. doi: 10.2196/79559 (PMC12779099; doi:10.2196/79559)
Supplement: Multimedia Appendix 4 [file jmir-v28-e79559-s004.pdf]

Education Action Plan (0-2Y acute phase)

Bathing — Cleaning. Once daily.

Ways

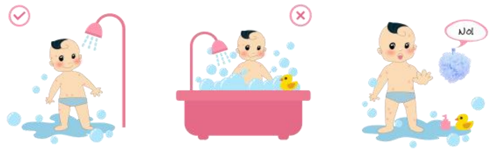

Shower, no soap base, do not wipe

Water temperature

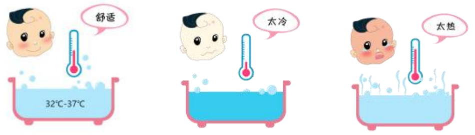

Water temperature 32-37°C

Emollient — Skin Barrier Repairation. At least twice a day

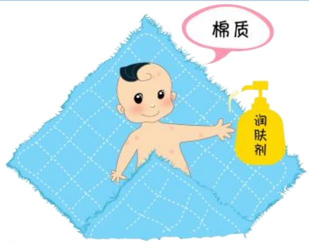

- **Pat off excess water with cotton products after bathing**
- **Apply emollient to the whole body within 5 minutes**
- **Daily Dosage ≧ 20g**
- **Product selection:**

✓ contains lipids and ceramides, unscented and colourless

Medication — Red skin rash without exudate. Combined emollient treatment.

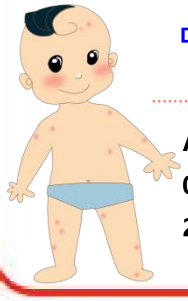

Denide Cream / Hydrocortisone Butyrate Cream

Once per night

Acute phase
0-2 years
2 weeks

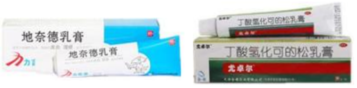

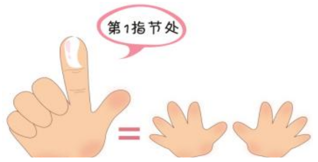

A strip of ointment as long as the first joint of your index finger is just right for covering an area about the size of your two palms.

Emergency — When to seek immediate medical attention and contact details

Pruritus scoring scale

1	2	3	4	5	6	7	8	9	10

Note: 1 least pruritic, 10 most severe

- **worsening itch: itch score ≧ 3**
- **Self-perceived exacerbation; requiring medical intervention**

Follow-up consultation details are available on the "Skin Care E Station" app.

Education Action Plan (>2Y acute phase)

Bathing   Cleaning. Once daily.

Ways

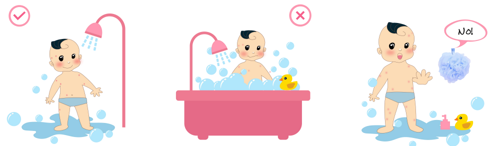

Shower, no soap base, do not wipe

Water temperature

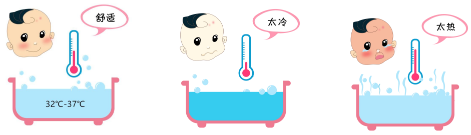

Water temperature 32-37°C

Emollient   Skin Barrier Repairation. At least twice a day

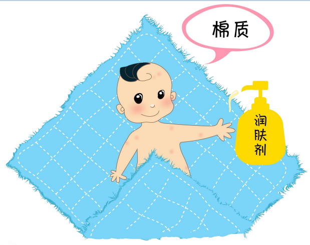

- **Pat off excess water with cotton products after bathing**
- **Apply emollient to the whole body within 5 minutes**
- **Daily Dosage ≧ 20g**
- **Product selection:**

✓ contains lipids and ceramides, unscented and colourless

Medication   Red skin rash without exudate. Combined emollient treatment.

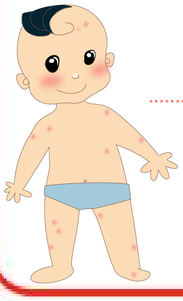

Mometasone Furoate Cream

Once per night

Acute phase
>2 years
2 weeks

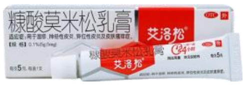

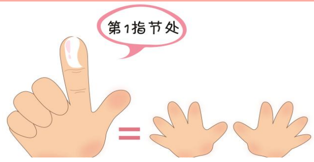

A strip of ointment as long as the first joint of your index finger is just right for covering an area about the size of your two palms.

Emergency   When to seek immediate medical attention and contact details

Pruritus scale scoring scale

1	2	3	4	5	6	7	8	9	10

Note: 1 least pruritic, 10 most severe

- **worsening itch: itch score ≧ 3**
- **Self-perceived exacerbation; requiring medical intervention**

Follow-up consultation details are available on the "Skin Care E Station" app.

Education Action Plan (0-2Y maintenance)

Bathing Cleaning. Once daily.

Ways

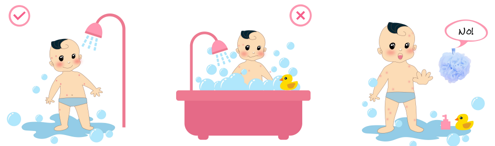

Shower, no soap base, do not wipe

Water temperature

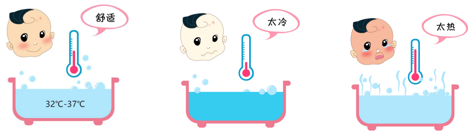

Water temperature 32-37°C

Emollient Skin Barrier Repairation. At least twice a day

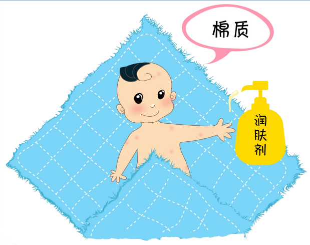

- **Pat off excess water with cotton products after bathing**
- **Apply emollient to the whole body within 5 minutes**
- **Daily Dosage ≧ 20g**
- **Product selection:**

✓ contains lipids and ceramides, unscented and colourless

Medication Red skin rash without exudate. Combined emollient treatment.

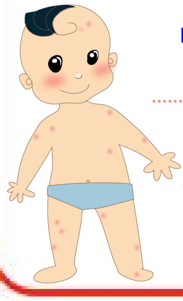

Denide Cream / Hydrocortisone Butyrate Cream

Twice per week

Maintenance
0-2 years
3 months

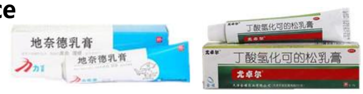

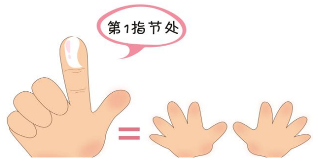

A strip of ointment as long as the first joint of your index finger is just right for covering an area about the size of your two palms.

Emergency When to seek immediate medical attention and contact details

Pruritus scoring scale

1	2	3	4	5	6	7	8	9	10

Note: 1 least pruritic, 10 most severe

- **worsening itch: itch score ≧ 3**
- **Self-perceived exacerbation; requiring medical intervention**

Follow-up consultation details are available on the "Skin Care E Station" app.

Education Action Plan (>2Y maintenance)

Bathing — Cleaning. Once daily.

Ways

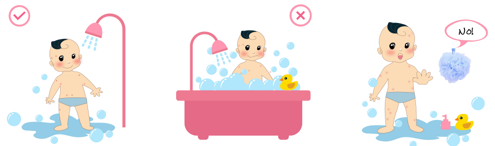

Shower, no soap base, do not wipe

Water temperature

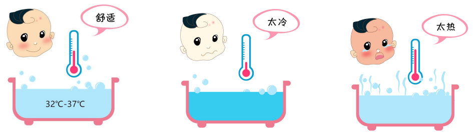

Water temperature 32-37°C

Emollient — Skin Barrier Repairation. At least twice a day

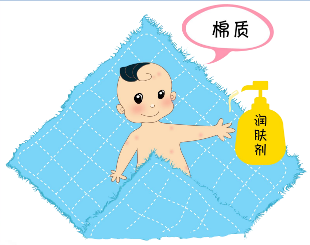

- **Pat off excess water with cotton products after bathing**
- **Apply emollient to the whole body within 5 minutes**
- **Daily Dosage ≧ 20g**
- **Product selection:**

✓ contains lipids and ceramides, unscented and colourless

Medication — Red skin rash without exudate. Combined emollient treatment.

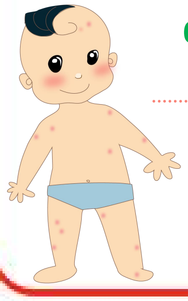

0.03% tacrolimus ointment

Twice per week

Maintenance

>2 years

3 month

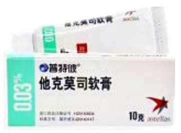

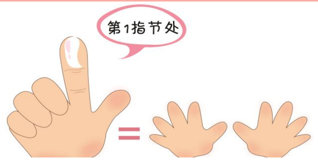

A strip of ointment as long as the first joint of your index finger is just right for covering an area about the size of your two palms.

Emergency — When to seek immediate medical attention and contact details

Pruritus scoring scale

1	2	3	4	5	6	7	8	9	10

Note: 1 least pruritic, 10 most severe

- **worsening itch: itch score ≧ 3**
- **Self-perceived exacerbation; requiring medical intervention**

Follow-up consultation details are available on the "Skin Care E Station" app.
