# Supplementary figures and images for: Smartphone-Based Digital Eczema Education Program for Atopic Dermatitis in Children Aged 0 to 6 Years: Multicenter, Randomized, Parallel Controlled Clinical Study
Source: J Med Internet Res. 2026 Jan 7;28:e79559. doi: 10.2196/79559 (PMC12779099; doi:10.2196/79559)

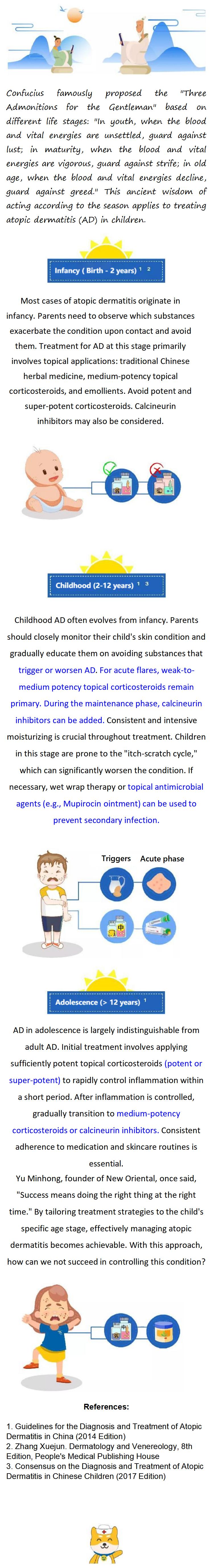

Supplement: Multimedia Appendix 5 [file jmir-v28-e79559-s005.png]

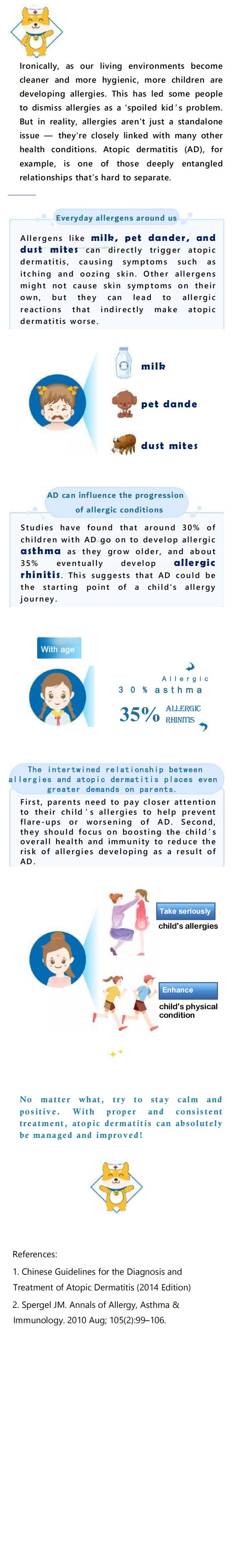

Supplement: Multimedia Appendix 6 [file jmir-v28-e79559-s006.png]
