# Supplementary material for: Smartphone-Based Digital Eczema Education Program for Atopic Dermatitis in Children Aged 0 to 6 Years: Multicenter, Randomized, Parallel Controlled Clinical Study
Source: J Med Internet Res. 2026 Jan 7;28:e79559. doi: 10.2196/79559 (PMC12779099; doi:10.2196/79559)
Supplement: Multimedia Appendix 8 [file jmir-v28-e79559-s008.pdf]

Nothing Wrong with Cleanliness, but Don't Overdo It

Pippi was very upset recently when his mother gave away his pet dog, White. The reason for this was that Pippi had developed atopic dermatitis, and his mother believed that having a pet might trigger a flare-up of the condition. To prevent this, Pippi's mother had started a strict cleaning routine every day. She also insisted that Pippi take two baths a day, once in the morning and once in the evening, and she restricted his outdoor activities. In fact, she didn't even let him play with his friends for fear that he might be exposed to germs. However, despite these efforts, Pippi's atopic dermatitis did not improve. Instead, his allergic rhinitis became more frequent, and the skin lesions deepened.

Pippi's father, unable to stand the situation any longer, commented on his wife's approach: “These practices of yours are an example of excessive cleaning, and they're not helping Pippi's atopic dermatitis at all. I did some research and found that in cities and developed countries, where hygiene is high and the quality of life is good, the incidence of atopic dermatitis in children is actually higher. Some studies have even suggested that frequent bathing can increase the risk of atopic dermatitis in children!”[1,2]

Skeptical, Pippi's mother decided to consult a doctor, who explained, “Frequent washing reduces children's exposure to pathogens, which is not ideal for developing a robust immune system. In fact, having a dog may even be a potential protective factor against atopic dermatitis.[3] Excessive cleaning is actually detrimental to your child's condition.”

Realizing she might have “overreacted”, Pippi's mother immediately made changes under the doctor's guidance. She reduced Pippi's baths to once a day, welcomed White back into the home, and encouraged Pippi to engage more with other children and play outdoors.

References

1. Schram ME, Tedja AM, Spijker R, Bos JD, Williams HC, Spuls PI. Is there a rural/urban gradient in the prevalence of eczema? A systematic review. Br J Dermatol. 2010 May;162(5):964-73. doi: 10.1111/j.1365-2133.2010.09689.x. Epub 2010 Mar 16. PMID: 20331459.
2. Sherriff A, Golding J; Alspac Study Team. Factors associated with different hygiene practices in the homes of 15 month old infants. Arch Dis Child. 2002 Jul;87(1):30-5. doi: 10.1136/adc.87.1.30. PMID: 12089118; PMCID: PMC1751130.
3. Pelucchi C, Galeone C, Bach JF, La Vecchia C, Chatenoud L. Pet exposure and risk of atopic dermatitis at the pediatric age: a meta-analysis of birth cohort studies. J Allergy Clin Immunol. 2013 Sep;132(3):616-622.e7. doi: 10.1016/j.jaci.2013.04.009. Epub 2013 May 24. PMID: 23711545.
