# Supplementary material for: Smartphone-Based Digital Eczema Education Program for Atopic Dermatitis in Children Aged 0 to 6 Years: Multicenter, Randomized, Parallel Controlled Clinical Study
Source: J Med Internet Res. 2026 Jan 7;28:e79559. doi: 10.2196/79559 (PMC12779099; doi:10.2196/79559)
Supplement: Multimedia Appendix 9 [file jmir-v28-e79559-s009.pdf]

Multimedia Appendix 9

1. Please mark the affected areas on the diagram below and upload corresponding photos.

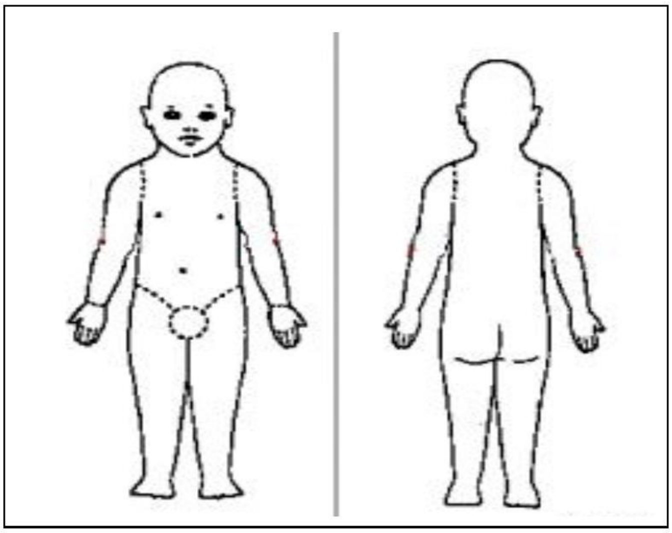

Front Back

2. Subjective Symptom Assessment (Please evaluate based on the condition over the last 3 days)

Pruritus Intensity	None	Aware of pruritus, occasional scratching	Frequent scratching but tolerable	Conscious of significant pruritus, frequent scratching	Severe pruritus, very frequent scratching	Persistent scratching, inability to sleep
	0	1 2	3 4	5 6	7 8	9 10
Sleep Loss	Unaffected	Mildly troubled, minimal impact on falling asleep	Difficulty falling asleep, but no early awakening	Significant difficulty falling asleep, noticeable early awakening	Severe difficulty falling asleep, waking 3-4 times per night	Near total inability to sleep
	0	1 2	3 4	5 6	7 8	9 10

Pruritus Symptom Score = _______ points
Sleep Loss Symptom Score = _______ points
