# Supplementary material for: Smartphone-Based Digital Eczema Education Program for Atopic Dermatitis in Children Aged 0 to 6 Years: Multicenter, Randomized, Parallel Controlled Clinical Study
Source: J Med Internet Res. 2026 Jan 7;28:e79559. doi: 10.2196/79559 (PMC12779099; doi:10.2196/79559)
Supplement: Multimedia Appendix 10 [file jmir-v28-e79559-s010.docx]

Multimedia Appendix 10. Comparison of changes in disease severity and quality of life scores between two groups over time (median (Q1,Q3))

|  |  | 4 week | 8 week | 12 week | 24 week | 36 week | 52 week |
| --- | --- | --- | --- | --- | --- | --- | --- |
| SCORAD | Digital education | 11.00 (4.50, 25.00) | 10.00 (4.00, 20.00) | 10.00 (1.00, 21.00) | 8.00 (0.00, 16.00) | 8.00 (0.00, 18.00) | 7.50 (0.00, 15.00) |
|  | Control | 11.00 (4.50, 24.00) | 11.00 (4.00, 23.00) | 11.00 (2.00, 23.00) | 8.00 (0.00, 16.00) | 9.00 (0.00, 15.00) | 8.00 (0.00, 17.00) |
|  | P value | 0.78 | 0.54 | 0.41 | 0.26 | 0.72 | 0.52 |
| PP-NRS | Digital education | 2.00 (1.00, 4.00) | 2.00 (1.00, 4.00) | 2.00 (1.00, 4.00) | 2.00 (1.00, 3.00) | 2.00 (1.00, 4.00) | 2.00 (1.00, 3.00) |
|  | Control | 2.00 (1.00, 4.00) | 2.00 (1.00, 4.00) | 2.00 (1.00, 4.00) | 2.00 (1.00, 3.00) | 2.00 (1.00, 3.00) | 2.00 (1.00, 3.00) |
|  | P value | 0.97 | 0.87 | 0.52 | 0.69 | 0.89 | 0.40 |
| POEM | Digital education | 4.00 (1.00, 9.50) | 3.00 (1.00, 7.00) | 2.00 (0.00, 7.00) | 2.00 (0.00, 6.00) | 2.00 (0.00, 6.00) | 2.00 (0.00, 6.00) |
|  | Control | 3.00 (1.00, 8.00) | 3.00 (1.00, 7.00) | 3.00 (1.00, 7.00) | 2.00 (0.00, 6.00) | 3.00 (0.00, 6.00) | 2.00 (0.00, 6.00) |
|  | P value | 0.80 | 0.71 | 0.22 | 0.66 | 0.36 | 0.99 |
| QoL | Digital education | 2.00 (1.00, 7.00) | 2.00 (1.00, 5.00) | 1.00 (0.00, 5.00) | 1.00 (0.00, 5.00) | 1.00 (0.00, 5.00) | 1.00 (0.00, 3.00) |
|  | Control | 2.00 (1.00, 6.00) | 2.00 (1.00, 6.00) | 2.00 (0.00, 5.00) | 1.50 (0.00, 5.00) | 1.00 (0.00, 4.00) | 1.00 (0.00, 3.00) |
|  | P value | 0.92 | 0.21 | 0.35 | 0.43 | 0.99 | 0.82 |
| DFI | Digital education | 2.00 (0.00, 8.00) | 1.00 (0.00, 7.00) | 0.00 (0.00, 6.00) | 0.00 (0.00, 5.00) | 0.00 (0.00, 5.50) | 0.00 (0.00, 4.00) |
|  | Control | 2.00 (0.00, 8.00) | 2.00 (0.00, 8.00) | 1.00 (0.00, 8.00) | 1.00 (0.00, 7.00) | 0.00 (0.00, 6.00) | 0.00 (0.00, 5.00) |
|  | P value | 0.79 | 0.14 | 0.35 | 0.27 | 0.68 | 0.46 |

This table presents the changes in disease severity scores (SCORAD, PP-NRS, and POEM) and quality of life scores (IDQOL/CDLQI and DFI) from week 2 (end of the acute treatment phase) to weeks 4, 8, 12, 24, 36, and 52 in both groups. Values are expressed as median (Q1, Q3). No significant differences were observed between the two groups at any of the predefined follow-up time points.

Abbreviations: SCORAD, scoring atopic dermatitis index; PP-NRS, peak-pruritus numerical rating scale; DFI, dermatitis family impact; POEM, patient oriented eczema measure; QoL, quality of life, children’s dermatology life quality index (CDLQI) for children, and infants’ dermatitis quality of life index (IDQOL) for infants; Q1, the first quartile; Q3, the third quartile.
